# Methods of the 2020 (Wave 1) International Tobacco Control (ITC) Malaysia survey

**DOI:** 10.18332/tid/146568

**Published:** 2022-03-31

**Authors:** Amer Siddiq Amer Nordin, Ahmad Syamil Mohamad, Anne C. K. Quah, Farizah Mohd Hairi, Anne Yee, Nur Amani Ahmad Tajuddin, Siti Idayu Hasan, Mahmoud Danaee, Susan C. Kaai, Matthew Grey, Pete Driezen, Geoffrey T. Fong, Mary E. Thompson

**Affiliations:** 1 Department of Psychological Medicine, Faculty of Medicine, Universiti Malaya, Kuala Lumpur, Malaysia; 2 Nicotine Addiction Research Group, University of Malaya Centre of Addiction Sciences, Universiti Malaya, Kuala Lumpur, Malaysia; 3 Department of Social and Preventive Medicine, Faculty of Medicine, Universiti Malaya, Kuala Lumpur, Malaysia; 4 Department of Psychology, University of Waterloo, Waterloo, Canada; 5 Department of Primary Care Medicine, Faculty of Medicine, Universiti Malaya, Kuala Lumpur, Malaysia; 6 School of Public Health Sciences, University of Waterloo, Waterloo, Canada; 7 Ontario Institute for Cancer Research, Toronto, Canada; 8 Department of Statistics and Actuarial Science, University of Waterloo, Waterloo, Canada

**Keywords:** sampling design, survey measures, response rate, cooperation rate, tobacco control policy

## Abstract

The ITC Malaysia Project is part of the 31-country ITC Project, of which the central objective is to evaluate the impact of tobacco control policies of the WHO Framework Convention on Tobacco Control (FCTC). This article describes the methods used in the 2020 International Tobacco Control (ITC) Malaysia (MYS1) Survey. Adult smokers and non-smokers aged ≥18 years in Malaysia were recruited by a commercial survey firm from its online panel. Survey weights, accounting for smoking status, sex, age, education, and region of residence, were calibrated to the Malaysian 2019 National Health and Morbidity Survey. The survey questions were identical or functionally similar to those used in other ITC countries. Questions included demographic measures, patterns of use, quit history, intentions to quit, risk perceptions, beliefs and attitudes about cigarettes, e-cigarettes, and heated tobacco products. Questions also assessed measures assessing the impact of tobacco demand-reduction domains of the FCTC: price/tax (Article 6), smoke-free laws (Article 8), health warnings (Article 11), education, communication and public awareness (Article 12), advertising, promotion, and sponsorship restrictions (Article 13), and support for cessation (Article 14). The total sample size was 1253 (1047 cigarette smokers and 206 non-smokers). Response rate was 11.3%, but importantly, the cooperation rate was 95.3%. The 2020 ITC MYS1 Survey findings will provide evidence on current tobacco control policies and evidence needed by Malaysian government regulatory agencies to develop new or strengthen existing tobacco control efforts that could help achieve Malaysia’s endgame, i.e. a tobacco-free nation by 2040.

## INTRODUCTION

### Background

An estimated 27000 Malaysians die from cigarette smoking each year^1^. In 2019, 21.3% of Malaysians aged ≥15 years smoked cigarettes, small reduction since 2015 (22.8%)^2^ and 2011 (23.1%)^3^. Smoking is much more prevalent among males than females in Malaysia. In 2019, 40.5% of males smoked compared with only 1.2% of females^1^. The use of e-cigarettes (ECs) is low, with only 4.9% of Malaysians aged ≥15 years reporting EC use in 2019. EC use is more common among Malaysians aged 20–24 years (14.7%)^1^.

Since the ratification of the World Health Organization (WHO) Framework Convention on Tobacco Control (FCTC) in 2005, Malaysia made several amendments to the Control of Tobacco Product Regulations (2004) to regulate smoke-free environments, price and tax, tobacco advertising, promotion and sponsorship, and tobacco packaging and labeling in the country^4,5^. These amendments include the implementation of pictorial health warnings and smoke-free public places, increasing cigarette taxes, and banning cigarette promotion and sponsorship by tobacco companies. In spite of this, there were no significant declines in smoking prevalence from 2011 to 2019. In addition, Malaysia, which has the largest e-cigarette market in Southeast Asia, has yet to regulate alternative tobacco and nicotine products^6^.

The International Tobacco Control (ITC) Malaysia Project is part of the 31-country International Tobacco Control Policy Evaluation (ITC) Project. The ITC Project’s central objective is to evaluate the impact of WHO FCTC policies. It is not possible to conduct controlled experiments to evaluate the effects of tobacco control policies because governments, not researchers, control the policy implementation. Thus the ITC Project across all 31 countries, including Malaysia, uses three major strategies to rigorously evaluate the effects of policies: 1) a quasi-experimental research design (i.e. ‘natural experiments’)^7^, in which one group exposed to a policy is compared to another, unexposed group; 2) the use of longitudinal cohort designs^8^ in which individuals are measured on the same key outcome variables over time^9,10^; and 3) the measurement of appropriate policy-specific variables that are conceptually close to the policy being evaluated and less likely to be affected by other factors. These innovative strategies, including other explanatory variables (covariates), are unparalleled in the study of population-level interventions and produce a research design with the potential to make strong inferences about policy impact^11-16^.

The ITC Malaysia Surveys have served as an evaluation system for measuring the impact of WHO FCTC policies implemented in Malaysia since 2005. The present work describes the methods of the International Tobacco Control Malaysia Wave 1 Survey (ITC MYS1), a new survey that is part of the longstanding ITC Project and the longstanding of the previous ITC Southeast Asia (SEA) Project. The ITC SEA Project had successfully conducted six survey waves in Malaysia and Thailand between 2005 and 2014. Findings from the previous ITC Malaysia Surveys have been published to provide evidence on the effectiveness of the Malaysia tobacco control policies implemented between 2005 and 2014, for example on smoke-free policies^17^, cigarette package warning labels^18^, price^19^, and on the FCTC MPOWER measures^20,21^. The previous ITC Malaysia Survey was focused on cigarette smoking and since 2014, the tobacco landscape of Malaysia has dramatically changed with the introduction of alternative tobacco or nicotine products such as heated tobacco products (HTPs) and e-cigarettes (ECs). Due to the longitudinal design of the original ITC SEA Project, and the length of time that passed since the last survey wave in Malaysia (2013–2014), concerns over respondent attrition required the development of a new ITC Malaysia Survey. The ITC MYS1 Survey consists of a completely new cohort of respondents and a new, web-based mode of interviewing. As a result, it is possible to compare data from the ITC MYS1 Survey with previous waves of the ITC SEA Project in cross-sectional analysis as well as anticipate additional waves, permitting longitudinal analysis, of the new ITC Malaysia Project.

### Objectives of the 2020 ITC Malaysia survey

The main objectives of the new ITC MYS1 Survey are to examine the patterns of smoking (consumption patterns, quitting behaviors), to examine the impact of tobacco control policies that were adopted after 2014 (i.e. after Wave 6 of the ITC SEA Project), and to examine the patterns of EC and HTP use among a cohort of smokers and non-smokers in Malaysia.

The findings of the ITC MYS1 Survey data will demonstrate the effectiveness of current tobacco control policies and provide evidence needed by Malaysian government regulatory agencies to develop new or strengthen existing tobacco control policies in the future.

## METHODOLOGICAL APPROACH

### Sampling design and method of recruitment

Respondents for the MYS1 Survey were recruited from a Malaysian online panel. Rakuten Insight is a leading online panel research firm in Asia that has conducted research for nearly 25 years. It has strict panel quality controls in place at multiple touchpoints (e.g. recruitment, registration). After registering for the panel, participants provide Rakuten Insight with key profiling and demographic information (e.g. age, sex, region of residence, education, smoking habits). This information allows Rakuten Insight to pre-identify participants that are the best match (e.g. aged ≥18 years, specific behavior such as smoking, etc.) for relevant surveys. This creates an optimal experience for participants as they receive surveys that fit their profile, and helps expedite fieldwork by not having to contact a much larger audience to find niche target groups. About 88% of Malaysians have access to the internet^22^. The Rakuten online panel was nationally representative of adult Malaysian cigarette smokers and non-smokers aged ≥18 years. The sampling design of the ITC MYS1 Survey ensured a sample that was broadly representative of the population of adult cigarette smokers and non-smokers having internet access.

Rakuten Insight’s online panel was used to construct the sampling frame. Sampling quotas for the survey were calculated using census estimates and published estimates of smoking prevalence from the 2015 National Health and Morbidity Survey for the population aged ≥15 years^2,23^. This information was used to generate overall quotas for non-smokers (including people who never smoked) by sex, as well as female smokers overall. No additional constraints were used in these three groups. For male smokers, the previous ITC Malaysia Wave 6 Survey (2012–2014)^24^ was used to estimate the proportion of smokers in each of three regions (eastern Peninsular Malaysia, western Peninsular Malaysia, and East Malaysia consisting of Sabah, Sarawak, and Labuan) by age group (18–39, 40–59, ≥60 years). Due to the low prevalence of smoking among women (1.4%), over-sampling of female smokers was employed^2,25^. The final targeted quota consisted of 1270 Malaysian adults aged ≥18 years (1070 smokers and 200 non-smokers)^25^. The sample sizes of smokers and non-smokers were determined by the estimated prevalence smoking in Malaysia^2,23^.

Rakuten Insight used demographic information provided by their panelists to identify and invite potential respondents to participate in the ITC MYS1 Survey. Rakuten sent an invitation to eligible panelists from their sample frame. This invitation included a link to the programmed ITC MYS1 Survey. The survey began with an information letter that explained the study’s purpose, information about ethics, and remuneration. Respondents were then presented with a consent question regarding their participation in the study. Those who provided consent to participate were screened according to study inclusion criteria. Once invited to the survey, panelists first completed a set of screening questions to ensure they were: 1) at least 18 years of age; 2) fit within an open quota cell based on their smoking status, region of residence, sex, and age; and 3) could be classified as a current smoker (at least monthly and had smoked 100 or more cigarettes in their lifetime) or a non-smoker who has not smoked 100 cigarettes in their lifetime, or former smoker who had not smoked at least five years previously (Table 2). Participants who qualified and completed a survey were remunerated with reward points equivalent to US$5 as a token of appreciation.

### Survey measures

All ITC surveys are developed using the conceptual framework^7^ and methods^8^ of the ITC Project, which has conducted extensive cohort surveys of tobacco use since 2002. The ITC survey questions, which include more than 150 measures directly related to policy impact, are designed to be identical or functionally equivalent across all ITC countries. This enables comparison of effects across countries. All surveys are publicly accessible on the ITC Project website (https://itcproject.org/surveys/).

The ITC MYS1 Survey contained questions assessing smoking history, current smoking behavior (including cigarettes and alternative tobacco products such as ECs and HTPs), addiction, cessation help, and questions about beliefs and knowledge of the harms of smoking. It also assessed the salience of health warning labels on tobacco packaging, perceptions of warning label effectiveness, and cognitive reactions to warning labels. Several items assessed the price of cigarettes, including the amount spent on the last purchase of cigarettes. Respondents were also queried about cigarette and EC advertising and promotion, their psychosocial beliefs, perceived risks, tobacco control regulations in their community, exposure to cigarettes and tobacco use at home and work, beliefs about the effectiveness of smoke-free laws, and several demographic measures. Table 1 lists key measures included in the ITC MYS1 Survey. Appropriate questions were asked of smokers and non-smokers after they were screened about their current use of cigarettes. For example, if non-smokers had never smoked and never used ECs or HTPs, they were not asked current use and quit attempt questions. However, questions on FCTC policies were asked of both smokers and non-smokers.

**Table 1:** Key measures of the 2020 ITC Malaysia survey

**Demographics:** sex, ethnicity, age, education, income, state of health
**Other personal moderators:** quitting history, nicotine dependence, levels of stress, including financial stress, depressed mood, time perspective, etc.
**Environmental moderators:** number of smokers and non-smokers in the household and in social network
**Policy-specific measures** of policies on products (cigarettes, e-cigarettes, heated tobacco products) and of FCTC policies:
**a) Article 6:** Price paid per unit of product, total weekly cost, product type/variant, purchasing unit, price perceptions.
**b) Article 14:** Use of cessation services and recall of advice, use of products and/or other medicines use in conjunction with professional assistance, advice on appropriateness of products use.
**c) Article 13:** Advertising/marketing: noticing advertisements and frequency in key channels (TV, print, internet), susceptibility to advertising, whether product advertising makes them think about cigarettes.
**d) Article 11:** Health warnings: salience and noticing of health warnings (if any), brand usage, perceived risks, perceived impact on product use; forgoing cigarettes because of the warnings.
**e) Article 8:** Smoke-free/vapor-free laws (and/or establishment policies): exposure to smoking/vaping in key venues, perceived impact on product use, reports on restrictions.
**f) Product availability:** Restrictions on access: perceived availability.
**g) Article 9:** Nicotine content, flavor and other product characteristics: nicotine content and flavors of product brands used, perceived addictiveness of products and cigarettes, and appeal of products.
**h) Article 12:** Awareness and recall of media campaigns on products and on anti-smoking themes.
**Psychosocial mediator variables:** Social norms for products, outcome expectancies for products, reasons for use, self-efficacy, and intentions to quit smoking; relative harmfulness, health concerns.
**E-cigarettes and tobacco use behaviors:** Key outcomes along with some of the variables for intermediary analyses. Use of e-cigarettes and other tobacco products: frequency of use, duration, and intensity of use (e.g. cigarettes per day); usual brand/type of product; quit attempts (smoking), duration of abstinence (smoking), product switching.

The survey content was first developed and finalized in English and translated into Malay and Chinese by professional translators. Both translations were reviewed by research team members who are bilingual and fluent in English and Malay or English and Chinese. Any issues that were identified were discussed and resolved to ensure that the Malay and Chinese translations met the research team’s standards for the highest possible degree of clarity and accuracy and have the closest equivalence to the English survey content, a method that is superior to traditional double translation methods^26^. Participants completed the online survey in one of these three languages. The full surveys in all three languages for ITC MYS1 Survey can be found at https://itcproject.org/surveys/malaysia/mys1-cohort2/.

## CASE STUDIES

**Figure 1:**
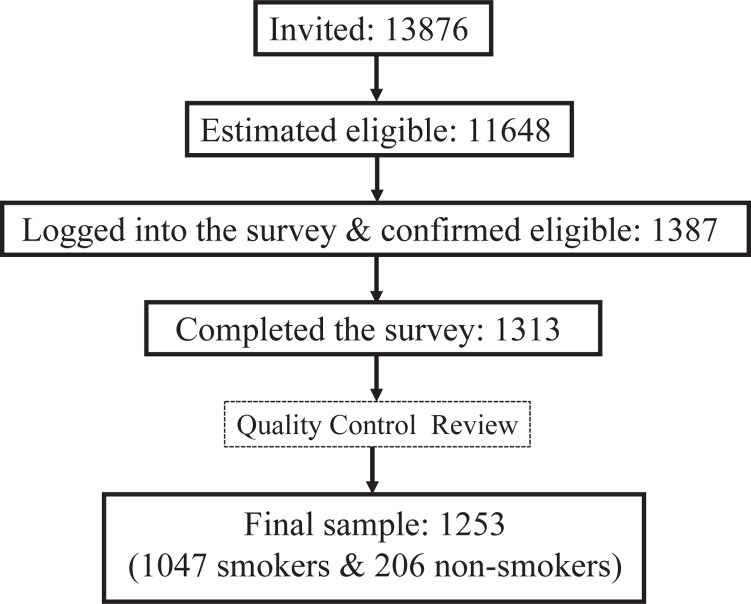
Sample recruitment of the 2020 ITC Malaysia Survey

A total of 13876 individuals (Figure 1) were invited by e-mail to participate in the survey. Of these, 11648 were eligible. Of eligible participants, 1387 logged into the survey, of which 1313 completed the survey^25^.

The final sample of the ITC MYSI1 Survey was 1253 (1047 smokers and 206 non-smokers)^25^. Table 2 presents the survey sample by user types, region of residence, sex, and age.

**Table 2:** ITC Malaysia Wave 1 Survey user group and sample size by region, sex, and age

*User group*	*Definition*	*Region Sex, Age (years)*	*Targeted n*	*Final n*
**Current smokers**	Smoked cigarettes at least monthly AND had smoked 100 or more cigarettes in their lifetime	Western and Eastern Peninsular Male (18–39)	620	588
		Western and Eastern Peninsular Male (40+)	200	206
		East Malaysia, Male (18+)	150	150
		All three regions Female (18+)	100	103
Total smokers			1070	1047
**Never/Non smokers**	Never smokers who had not smoked 100 cigarettes in their lifetime OR former smokers who had quit for a period longer than 5 years	Male (18+)	77	80
		Female (18+)	123	126
Total non-smokers			200	206
**Grand total**			**1270**	**1253**

### Response and cooperation rates

Two key survey statistics were computed: 1) the response rate, defined as the number of completed interviews divided by the estimated number of participants invited to the survey at a time when their quota was ‘open’ and they were eligible to participate [the American Association for Public Opinion Research (AAPOR) Response Rate 4 (RR4)]^27^; and 2) the cooperation rate, defined as the number of completed interviews divided by the number of participants who entered the survey and proceeded as far as confirming their eligibility [AAPOR Cooperation Rate 4 (COOP4)]27. For the ITC MYS1 Survey, the response rate was 11.3% and the cooperation rate was 95.3%. It should be noted that the response rate includes in the denominator people who were invited but may never have seen or noticed the invitation. Thus, the response rate underestimates (to an unknown extent) the proportion who would have responded to an invitation received27. Because of this, the cooperation rate may be a better indicator of the potential sampling bias due to the content of the survey (tobacco use). The very high cooperation rate is a sign that the potential bias due to refusal on the basis of survey content was very low because eligibility was confirmed following consent to participate prior to the interview.

### Quality control

A pilot study was conducted from 5–7 February 2020 to ensure that the data collection procedures worked correctly. These pilot data were reviewed by both Rakuten and the research team to identify potential problems and to rectify them prior to the official launch. Throughout the fieldwork, Rakuten Insight closely monitored the survey activity and provided a link to enable real-time monitoring of survey activity.

Two measures were used to identify survey records of poor data quality: time taken to answer each question in seconds (SecperQ), and the percentage of ‘Refused’ or ‘Don't know’ responses (%RDK). Poor data quality records were defined as having very low SecperQ and/or high %RDK^25^. Extreme values occurred for both of these measures, i.e. SecperQ times of less than 1.93 seconds per question, which by published estimates is insufficient to read the question, and %RDK responses for more than 84% of the questions completed by the respondent^25^. In the ITC MYS1 Survey, smokers and non-smokers were examined separately because they were required to answer different sets of questions. Detailed criteria for assessing poor data quality are described in a technical report^25^.

### Data protection

Survey responses are stored in a computer file; the only identifying information is the respondent’s survey ID number. Respondent names are not attached to the data file and are held exclusively by the survey firm, Rakuten Insight. The data contain references to the unique IDs only.

### External data sharing

Researchers who are interested in analyzing study data will be required to apply for approval by submitting a request to the International Tobacco Control Data Repository at the University of Waterloo, Canada. Further information about the data sharing policy can be found on the ITC Project’s website (https://itcproject.org/request-data-form/). For external researchers, the data are available two years after the cleaned and weighted data are released to the ITC Malaysia research team.

### Survey weights

Two sets of survey weights were computed for the ITC MYS1 data: inflation weights and analytic weights. The inflation weight for a given respondent is interpreted as the number of people in the population represented by that respondent. Because it was not possible to estimate the probability of inclusion for respondents, each respondent was assigned an initial weight of 1. These initial weights were then inflated and calibrated using a raking algorithm so that within specified categories they would sum to population totals estimated from the Malaysian National Health and Morbidity Survey of 2015^2,23^. These published totals yielded estimated population sizes for smokers and non-smokers, separately by sex, age group, ethnicity, education, and region of residence. Complete details regarding the computation of inflation weights are available in a technical report^25^.

Analytic weights (or ‘rescaled’ weights) were also calculated. These weights are the inflation weights rescaled to sum to the final sample sizes within: 1) smokers, and 2) non-smokers who completed the survey. The rescaled weights are typically used for regression analysis and other inferential statistical methods. For efficiency of estimation, it is advised to include the weighting variables (sex, age group, ethnicity, education, and region of residence) as covariates in regression models.

### Demographic characteristics of respondents

Table 3 presents the demographic characteristics of the sample by smoking status. Unweighted percentages highlight the distribution of respondents within each smoking category whereas weighted estimates represent the population distribution within each smoking category (daily smokers, non-daily smokers, and non-smokers). Generally, unweighted and weighted percentages are similar within each the three smoking categories. However, the inflation weights exert a large influence in some cases to ensure sampled respondents represent the overall population of smokers and non-smokers in Malaysia. For example, 35.0% of non-smoking respondents were of Malay ethnicity, while Malays represent 62.7% of the non-smoking population.

**Table 3:** Demographic characteristics of respondents participating in the ITC Malaysia Wave 1 Survey

*Characteristics*	*Daily*			*Non-daily*			*Non-smoker*			*Overall*		
	*n*	*U %*	*W %*	*n*	*U %*	*W %*	*n*	*U %*	*W %*	*n*	*U %*	*W %*
Region*												
Western Peninsular Malaysia	401	45.2	41.4	70	43.8	40.3	105	51.0	44.1	576	46.0	43.5
Eastern Peninsular Malaysia	188	21.2	21.5	31	19.4	25.2	43	20.9	24.3	262	20.9	23.7
Kuala Lumpur + Putrajaya	159	17.9	12.6	38	23.8	18.0	29	14.1	12.5	226	18.0	12.6
East Malaysia	139	15.7	24.5	21	13.1	16.5	29	14.1	19.2	189	15.1	20.1
**Sex**												
Male	813	91.7	97.8	131	81.9	93.6	80	38.8	37.4	1024	81.7	50.9
Female	74	8.3	2.2	29	18.1	6.4	126	61.2	62.6	229	18.3	49.1
**Age** (years)												
18–24	122	13.8	14.0	22	13.8	11.6	36	17.5	17.7	180	14.4	16.8
25–39	538	60.7	50.7	97	60.6	53.2	114	55.3	55.7	749	59.8	54.6
40–54	190	21.4	30.2	33	20.6	26.5	42	20.4	20.0	265	21.1	22.2
≥55	37	4.2	5.1	8	5.0	8.7†	14	6.8	6.6†	59	4.7	6.4
**Marital status**												
Single	295	33.3	33.5	61	38.1	29.8	89	43.2	36.0	445	35.5	35.3
Married/living with partner	540	60.9	58.8	91	56.9	61.6	113	54.9	62.6	744	59.4	61.8
Separated/divorced/widowed/not reported	52	5.9	7.8	8	5.0	8.6†	4	1.9	1.4†	64	5.1	2.9
**Ethnicity**												
Malay	480	54.1	57.6	67	41.9	55.1	72	35.0	62.7	619	49.4	61.4
Chinese	265	29.9	13.8	76	47.5	25.4	120	58.3	28.9	461	36.8	25.8
Other	135	15.2	27.0	13	8.1	13.7	14	6.8	8.5†	162	12.9	12.3
Not reported	7	0.8	1.6†	4	2.5	5.8†	0	–		11	0.9	0.5†
**Religion**												
Islam	515	58.1	67.4	71	44.4	62.1	80	38.8	68.0	666	53.2	67.7
Christianity	113	12.7	16.7	15	9.4	8.3†	23	11.2	7.4	151	12.1	9.3
Buddhism	155	17.5	8.0	41	25.6	14.7	73	35.4	17.9	269	21.5	15.9
Other/Not reported	59	6.7	4.4	18	11.3	6.7	21	10.2	4.5	98	7.8	4.5
Not religious	45	5.1	3.5	15	9.4	8.2†	9	4.4	2.1†	69	5.5	2.5
**Education level**												
≤Upper secondary	316	35.6	49.0	36	22.5	33.6	52	25.2	29.6	404	32.2	33.6
Diploma/certificate	257	29.0	35.5	43	26.9	41.6	62	30.1	41.8	362	28.9	40.6
Bachelor’s degree or higher	310	34.9	14.9	80	50.0	22.4	90	43.7	26.7	480	38.3	24.2
Not reported	4	0.5	0.7†	1	0.6	2.3†	2	1.0	1.9†	7	0.6	1.6†
**Employment status**												
Employed full/part-time	772	87.0	83.6	136	85.0	82.8	147	71.4	67.0	1055	84.2	70.7
Unemployed	34	3.8	4.1	8	5.0	5.0†	8	3.9	4.4†	50	4.0	4.4†
Retired/pension/student/home duties/other	72	8.1	11.2	15	9.4	11.5†	49	23.8	27.4	136	10.9	23.8
Not reported	9	1.0	1.2†	1	0.6	0.7†	2	1.0	1.2†	12	1.0	1.1†
**Occupation**												
Professional	219	24.7	15.0	54	33.8	21.0	57	27.7	20.5	330	26.3	19.5
Administrative	119	13.4	9.3	27	16.9	13.8	39	18.9	16.5	185	14.8	15.0
Service	177	20.0	23.0	30	18.8	24.0	25	12.1	16.4	232	18.5	17.9
Skilled	143	16.1	18.7	17	10.6	11.0	15	7.3	7.8	175	14.0	10.0
Unskilled/agriculture	34	3.8	5.0	5	3.1	5.2†	6	2.9	5.6†	45	3.6	5.5†
Pensioner/student/unemployed/domestic duties/other	165	18.6	24.3	22	13.8	19.8	53	25.7	29.4	240	19.2	28.2
Not reported	30	3.4	4.7	5	3.1	5.1†	11	5.3	3.7†	46	3.7	4.0
**Cigarettes/day (**smokers only)												
≤10	487	55.6	51.3		S			N/A		639	62.1	56.6
11–20	342	39.0	42.6							343	33.3	38.0
≥21	47	5.4	6.1							47	4.6	5.4
Mean	876	12.14	13.11	153	1.52	1.54		N/A		1029	10.64	11.83

U %: unweighted percentage. W %: weighted percentage.

* Region: Western Peninsular Malaysia: Perlis, Kedah, Penang, Perak, Selangor, Negeri Sembilan, and Melaka. Eastern Peninsular Malaysia: Kelantan, Terengganu, Pahang, and Johor. East Malaysia: Sabah, Sarawak, and Labuan.

† High sampling variability relative standard error >30%, interpret with caution.

S: suppressed due to small cell sizes in some categories. N/A: not applicable. All weighted percentages were estimated using the inflation weights, except cigarettes smoked/day where means and percentages were estimated using the rescaled weights.

Most smokers in Malaysia are male (97.8%) while fewer non-smokers were male (37.4%) than female (62.6%). The age distribution was similar across smoking categories, although a slightly smaller percentage of smokers were aged 25–39 years (50.7%) than non-smokers (55.7%). A greater percentage of smokers had an upper secondary school education or less (49.0%) than non-smokers (29.6%). Finally, a much greater percentage of smokers were employed full-time (83.6%) than non-smokers (67.0%).

## DISCUSSION

The sampling and data collection methods of the 2020 ITC MYS1 Survey were designed to re-establish an evidence system in Malaysia that can be used to measure and understand tobacco use and to evaluate the impact of FCTC tobacco control policies. The methods are consistent with 30 other ITC countries across the global ITC Project. Using the same protocols enables comparability of analyses across ITC countries^7,8,11-16^.

The final sample consisted of 206 non-smokers and 1047 smokers, of whom 103 were female and 944 were male^25^. These subsamples are not simple random samples, because they are further stratified by the quotas (e.g. age and region of residence for male smokers). Moreover, although respondents came from a high quality commercial online panel, initial recruitment into that panel was not probability-based. If the subsamples were simple random samples, we could estimate corresponding sub-population proportions with standard error at most 0.5 * √(1/n), which is 0.035 for the non-smoker sub-population, 0.049 for the female smoker sub-population, and 0.016 for the male smoker sub-population.

Stratification into quotas increases the precision of estimation, as imposition of the quotas improves the representativeness of each sub-sample. Because panel recruitment was not probability-based, some biases may exist. In particular, sub-samples were not fully representative of the sub-populations on some characteristics, for example, access to and familiarity with the internet, age, language, and ethnicity.

### Limitations

The ITC1 MYS Survey holds great potential to provide evidence on the impact of the FCTC in Malaysia, for policies already enacted and policies that will be implemented in the future. However, with a limited budget, only one wave of this cohort study has been conducted to date. Efforts are currently being made to secure funding to conduct additional follow-up surveys.

## CONCLUSIONS

The new ITC Malaysia Project has strong potential to provide important evidence on the effectiveness of Malaysia’s current and future tobacco control policies. To date, tobacco control policy formulation and implementation has almost exclusively focused on cigarettes. The new ITC Malaysia Project is timely as Malaysia is working towards reducing smoking prevalence to 15% or less by 2025 and to become a tobacco-free country by 2045^28^. The upcoming proposal of a new Tobacco Control Act in 2022 will strengthen regulations on cigarettes and include new regulations on alternative tobacco products^29^.

## Data Availability

The data supporting this research are available from the authors on reasonable request.
